# Extracellular vesicles and amyotrophic lateral sclerosis: from misfolded protein vehicles to promising clinical biomarkers

**DOI:** 10.1007/s00018-020-03619-3

**Published:** 2020-08-16

**Authors:** Delia Gagliardi, Nereo Bresolin, Giacomo Pietro Comi, Stefania Corti

**Affiliations:** 1 Dino Ferrari Centre, Neuroscience Section, Department of Pathophysiology and Transplantation (DEPT), University of Milan, Via Francesco Sforza 35, 20122 Milan, Italy; 2 Neurology Unit, Foundation IRCCS Ca’ Granda Ospedale Maggiore Policlinico, Via Francesco Sforza 35, 20122 Milan, Italy

**Keywords:** Extracellular vesicles, Amyotrophic lateral sclerosis, Prion-like properties, Biomarkers, Neurodegenerative disorders, Therapeutics

## Abstract

Extracellular vesicles (EVs) are small reservoirs of different molecules and important mediators of cell-to-cell communication. As putative vehicles of misfolded protein propagation between cells, they have drawn substantial attention in the field of amyotrophic lateral sclerosis (ALS) and other neurodegenerative disorders. Moreover, exosome-mediated non-coding RNA delivery may play a crucial role in ALS, given the relevance of RNA homeostasis in disease pathogenesis. Since EVs can enter the systemic circulation and are easily detectable in patients’ biological fluids, they have generated broad interest both as diagnostic and prognostic biomarkers and as valuable tools in understanding disease pathogenesis. Here, after a brief introduction on biogenesis and functions of EVs, we aim to investigate their role in neurodegenerative disorders, especially ALS. Specifically, we focus on the main findings supporting EV-mediated protein and RNA transmission in ALS in vitro and in vivo models. Then, we provide an overview of clinical applications of EVs, summarizing the most relevant studies able to detect EVs in blood and cerebrospinal fluid (CSF) of ALS patients, underlying their potential use in aiding diagnosis and prognosis. Finally, we explore the therapeutic applications of EVs in ALS, either as targets or as vehicles of proteins, nucleic acids and molecular drugs.

## Introduction

Extracellular vesicles (EVs) are cell-derived reservoirs of different molecules, proteins and factors, providing a crucial mean of communication and material exchange between adjoining and distant cells. They have recently gained a wide relevance in the field of amyotrophic lateral sclerosis (ALS), and neurodegenerative disorders overall, both as carriers of misfolded pathological proteins and non-coding regulatory RNAs and as vehicles of interplay between neurons and glial cells within central nervous system (CNS). Moreover, since detectable in patients’ biological fluids, EVs have become attractive clinical tools with wide applications in diagnosis, prognosis and therapeutics.

## Extracellular vesicles: biology and functions

EVs are encapsulated particles enriched in several molecules, including membrane and cytoplasmic proteins, lipids and nucleic acids. As shuttles, they deliver molecular cargoes to recipient cells within a biological system, representing one of the most relevant mechanisms of intercellular communication both in physiological and pathological processes.

EVs may be classified according to size, biogenesis and biochemical composition and are subdivided into microvesicles (MVs) and exosomes [1]. MVs range from approximately 100–150 to 1000 nm in diameter and are shed by budding of plasma membrane. Unlike exosomes, their production is mainly calcium-dependent; other factors like cellular stress and immune response may trigger MV release. Conversely, exosomes have diameters between 30 and 150 nm, sometimes overlapping with MVs, and are generated by inward budding of endosomal multivesicular bodies (MVBs) [2]. Depending on the cargo they are delivering, MVBs can either fuse with lysosome entering the protein degradation pathway or be transported to the plasma membrane and released by exocytosis. Exosomes are then exported into the extracellular space and are able to enter the body circulation and to cross the blood brain barrier (BBB) [3]. Further, they are detectable in biofluids like cerebrospinal fluid (CSF), blood and urine [4, 5], so they have acquired great relevance in the study of neurological disorders.

Surrounded by a phospholipid bilayer, EVs protect molecules from degradation and enable their delivery to adjacent and distant recipient cells. Donor cell type and physiological state determine the cargo and the composition of EVs and define their primary function. First, EVs can carry biologically active mediators involved in many biological processes, aimed to maintain homeostasis [6]. For instance, morphogens act stimulating development and cellular growth and differentiation [7], molecules released by tumor cell-derived EVs provoke angiogenesis, proliferation and metastasis [8], and bioactive lipids promote cytokine secretion, which in turn may induce chemotaxis, inflammation or apoptosis. Additionally, they might function as vehicles for elimination of unnecessary materials, playing a neuroprotective role. Similarly, exosomes have been shown to deliver active enzymes to assist degradation of potential toxic proteins [9]. Further, other than transporting useful proteins, EVs enable genetic material exchange between cells. Indeed, messenger RNAs (mRNAs) can be send to the recipient cells, where they are translated into functional proteins; besides, micro-RNAs (miRNAs) and long non-coding RNAs (lnRNAs) delivered by EVs may regulate gene expression in recipient cells [10].

All cell types in the CNS can release EVs, including neurons, astrocytes, oligodendrocytes and microglia [11]. In vitro studies [12, 13] have reported the high contribution of neuronal EVs, particularly exosomes, into synaptic activity modulation, since they can regulate the neurotransmitter release and the amount of post-synaptic receptors, as well as the neuronal excitability. MVs released by microglia are mainly involved in cytokine secretion, other than enhancing excitatory transmission [14], while microglial exosomes likely play a role in immunity [15]. Conversely, exosomes released by oligodendroglia are involved in neuronal firing rate and survival, since they can induce the transcription of genes implicated in stress response [16]. Astrocytes release a variety of EVs and according to their cargoes may mediate neurogenesis and synaptogenesis, myelination and neuroprotection or immunoregulatory and anti-apoptotic effects [17]. Importantly, astrocyte-derived MVs contain glutamate transporters and provide to their subcellular localization [18], consistently with astrocyte function of scavengers of extracellular glutamate.

Interestingly, a longitudinal study aimed at evaluating age-related physiological changes in plasma EVs has shown a decrease in EV concentration due to higher B-cell-mediated internalization and variation in protein composition with age [19]. Specifically, reduced levels of apoptotic markers (e.g. p-53, caspase-3) and enrichment in proteins involved in tumorigenesis and metastasis have been found in older patients, suggesting that EV characterization may provide relevant insights into paraphysiological processes like aging and potentially on age-related chronic diseases.

## Extracellular vesicles in neurodegenerative disorders

Neurodegenerative disorders have been increasingly recognized as proteinopathies, namely diseases resulting from abnormal deposition of misfolded and/or aggregated proteins. A growing body of evidence has pointed at a prion-like propagation as a possible mechanism underlying cell-to-cell protein transmission [20, 21]. According to this prion-like hypothesis, misfolded proteins can be transferred to healthy cells, induce their endogenous counterpart to misfold and lead to the amplification of these pathological seeds, prompting the onset and the progression of neurodegenerative disorders [22]. The spreading of the seeds between anatomically connected brain areas may occur through endocytosis/pinocytosis [23], tunneling nanotubes [24] and EV-mediated secretion [25].

A number of proteins, including Aβ, α-synuclein and tau have been found to colocalize with exosomes [26–31], supporting the role of EVs as potential drivers of pathological protein transmission and consequent neurodegeneration. Conversely, exosomes delivering proteolytical enzymes active against extracellular Aβ would suggest their biological role in preventing neurodegeneration [9]. Moreover, there is also counteractive evidence suggesting that pathological proteins can be transmitted as free proteins and seed propagation may occur through mechanisms other than exosome-based secretion [32].

In addition, the concept of neurodegenerative disorders as “non-cell autonomous” diseases is progressively gaining round, thus, in this context EV-mediated cell–cell communication, as well as neuronal–glial interplay, may play a crucial role in disease pathogenesis [33]. Neuroinflammation has been suggested as putative mechanism in neurodegenerative disorders. In this context, given their ability to produce immunomodulatory cytokines, glial cells, and especially microglia, are able to sense neuronal activity and to influence synaptic transmission in response to inflammatory stimuli.

Indeed, microglia-derived EVs generated in inflammatory conditions harbor different cargoes (pro-inflammatory cytokines and miR-155) compared to EVs derived from homeostatic microglia [34]. Other than inducing detrimental effects on neurons, the production of inflammatory mediators and reactive oxygen species (ROS) leads to further microglial activation, generating a vicious cycle of inflammation and neurodegeneration. In a recent study [35], MVs from inflammatory microglia were enriched in a set of miRNAs involved in regulation of key synaptic protein expression. Prolonged exposure to miR-146a-5p-containing EVs, which inhibits the expression of presynaptic synaptotagmin1 and postsynaptic neuroligin1, induced significant dendritic spine loss and decrease in density and strength of excitatory synapses. Consistently, astrocyte-derived exosomes from AD patients display higher expression of TNFα, IL-1β and IL-6 and higher level of complement proteins respect to controls [36]. In PD, microglial-derived EVs release several pro-inflammatory molecules in response to oxidative stress, causing harmful effects on dopaminergic neurons [37].

Notably, neuroprotective effects of glial cell-derived EVs in neurodegenerative disorders have been described as well, as shown by secretion of anti-inflammatory astrocyte-derived factors in parkinsonian brains [37]. In addition, exosomes isolated by adipose stem cells (ASCs) display neuroprotective and anti-apoptotic effects on an in vitro model of ALS [38]. Taken together, these findings support a dual role for EV-mediated glial-neuron crosstalk in neurodegenerative disorders.

Therefore EVs, particularly exosomes, have generated significant interest in the field of neurodegenerative disorders, given their application both in understanding disease pathogenesis and in improving diagnosis, prognosis and therapeutics of neurologic disorders.

ALS is a devastating disorder due to degeneration of upper and lower motor neurons (MNs). The pathological hallmark underlying most of ALS cases and half of the cases of frontotemporal lobar degeneration (FTLD) is the deposition of nuclear and cytoplasmic inclusions of TAR DNA-binding protein 43 (TDP-43) and phosphorylated-TDP-43 (pTDP-43) [39]. ALS-affected patients experienced focal weakness and atrophy, with subsequent clinical involvement of neighboring districts. The spreading of symptomatology to contiguous regions seems to trace clinically the transmission of misfolded proteins occurring on a molecular level between adjoining cells and within interconnected CNS areas.

Here, we aim to investigate the main functions of EVs in ALS, highlighting their role in disease pathogenesis. In particular, we summarize in vitro and in vivo evidence of EV-mediated prion-like misfolded protein propagation in ALS, focusing on the proteins encoded by the main ALS-causative genes, i.e. *superoxide dismutase 1 (SOD1), TARDBP, Fused-in-sarcoma (FUS) and Chromosome 9 open reading frame 72 (C9orf72).* Then, since alteration of RNA homeostasis is a crucial process in ALS, we provide an overview on EV-mediated non-coding RNA transmission and we point out the contribution of miRNAs into the regulation of gene expression in ALS. Finally, we explore the various applications of EVs in the field of motor neuron disorders and we underline their relevance as tools for pathogenesis comprehension, biomarkers in disease diagnosis and prognosis, as well as potential vehicles or targets for therapeutic approaches.

## Evidence of prion-like EV-mediated misfolded protein propagation in amyotrophic lateral sclerosis

Several proteins encoded by genes causative of ALS and involved in disease pathogenesis have been characterized over the past decades. Recent evidence suggests that many of these proteins have been retrieved in EVs and travel between neuronal and glial cells and within different brain areas, contributing to disease spreading and propagation [40]. Among them, particular mention has to be done to SOD1, TDP-43, FUS and dipeptide-repeat proteins (DPRs) (Table 1).

**Table 1:** Proteins with relevance for ALS identified in extracellular vesicles from in vitro and in vivo models and ALS patients

Protein	Study	Extracellular vesicles	Sample/model	Main finding
In vitro and in vivo studies				
SOD1	[43]	Exosomes	Mouse MN-like NSC-34 cells	Possible protective role of SOD1-containing exosomes against ROS
SOD1	[44]	Exosomes	*SOD1* overexpressing astrocytes	Astrocyte-derived exosomes contribute to neuronal toxicity
SOD1	[47]	Exosomes	Mouse MN-like NSC-34 cells	SOD1 is transmitted from cell to cell through exosomes and misfolding native SOD1 is efficiently perpetuated in naïve cells
SOD1	[48]	Exosomes	Rat microglial cells	Microglial cells release SOD1-containing exosomes and are toxic to neurons
SOD1	[49]	Exosomes and MVs	*SOD1* transgenic mouse	SOD1 is secreted in vivo in EVs derived from astrocytes and neurons
TDP-43	[50]	Exosomes	Human neuroblastoma cells	Phosphorylated TDP-43 aggregates can propagate from cell-to-cell via exosomes
TDP-43	[53]	Exosomes	U251 cells	TDP-43-containing exosomes from CSF from ALS/FTD patients has prion-like transmissible properties in vitro
TDP-43	[55]	Exosomes and MVs	HEK-293 cells and primary mouse neurons	Intracellular transmission and seeding properties
TDP-43	[56]	Exosomes	Neuro2a cells and TDP-43 transgenic mouse	Cytoplasmic TDP-43 localization in vitro*;* possible contribution in TDP-43 neuronal clearance in vivo
FUS	[58]	Exosomes	SH-SY5Y and N2A cells	FUS secretion in *FUS*-overexpressing cells
DPRs	[61]	Exosomes	iPSC-derived MNs from C9orf72-related ALS patients	Cell-to-cell DPR transmission
Human studies				
SOD1, TDP-43 and FUS	[82]	MVs	Plasma of sALS patients	Elevated levels in ALS compared to controls
TDP-43	[54]	Exosomes	CSF of ALS patients	CSF brain-derived exosomes contain TDP-43, reflecting neuropathology

*ALS* amyotrophic lateral sclerosis, *CSF* cerebrospinal fluid, *DPRs* dipeptide-repeat proteins, *EVs* extracellular vesicles, *FTD* frontotemporal dementia, *FUS* fused-in-sarcoma, *iPSC* induced pluripotent stem cell, *MN* motor neuron, *MVs* microvesicles, *ROS* reactive oxygen species, *sALS* sporadic ALS, *SOD1* superoxide dismutase, *TDP-43* TAR DNA-binding protein 43

SOD1 is a cytosolic and mitochondrial enzyme involved in clearance of superoxide molecules. Misfolded SOD1 aggregates have been reported in spinal MNs and glial cells of familial ALS (fALS) cases harboring *SOD1* mutations and of some sporadic forms (sALS) [41, 42]. Interestingly, SOD1 has been the first ALS-associated protein retrieved in EVs. In particular, SOD1 has been found in association with exosomes derived from mouse motor neuron-like NSC-34 cells overexpressing wild-type and mutated *SOD1* [43]. The authors speculate about the possible protective role of SOD1-positive exosomes against the physiological production of ROS outside the plasma membrane. A few years later, *SOD1* overexpressing astrocytes have been shown to release mutant SOD1-containing exosomes able to prompt MN death [44], thus suggesting that astrocytes and EVs may contribute to neuronal toxicity and disease spreading. After that, seed conversion of wild-type SOD1 by its misfolded counterpart has been demonstrated in intracellular compartments [45, 46], Grad and colleagues have provided evidence that misfolded native and mutated SOD1 can be transmitted from cell to cell through exosome-dependent and independent fashion and that propagated misfolding of native SOD1 may be efficiently perpetuated into naïve cells [47]. Further, cell exposure to conditioned media derived from *SOD1* overexpressing HEK293 cells induces intracellular accumulation of misfolded SOD1, while this does not occur after conditioned media depletion from EVs [47]. SOD1 overexpressing microglial cells have been found to release SOD1-containing exosomes as well [48]. Besides, they have shown toxicity in co-culture with primary neurons, which can be alleviated by treatment with the autophagy-inducer trehalose [48]. These findings strongly support the role of EVs in misfolded protein propagation following a prion-like fashion. Recently, a study performed in *SOD1* transgenic mouse model has shown that misfolded SOD1 protein is enriched in EVs released by neurons and astrocytes [49].

*TARDBP* encodes for mainly nuclear RNA-binding protein (RBP) involved in splicing and RNA metabolism. Aggregates of phosphorylated and ubiquitinated TDP-43 are the main neuropathological findings in brains from ALS patients [39]. C-terminal fragment (CTF) of TDP-43 contains a prion-like domain (PrLD) which allows protein–protein interaction and is able to induce seed-dependent aggregation in vitro [50]. Most of *TARDBP* disease-causing mutations involve CTF [51] and induce TDP-43 cytoplasmic mislocalization and pathological aggregate formation [52], resulting in RBP aberrant sequestration and impairment of RNA metabolism and proteostasis.

Exposure of *TDP-43*-expressing human neuroblastoma cells to insoluble TDP-43 from brains of ALS and frontotemporal dementia (FTD) patients results in template aggregation; moreover, pTDP-43 aggregates can be propagated from cell to cell at least partly via exosomes [50]. TDP-43 enrichment in EV fraction of CSF from ALS/FTD patients [53, 54] also supports the role of EVs in disease propagation. Moreover, CSF enriched in TDP-43-containing exosomes may act as seed in vitro, since it is able to prompt accumulation of toxic TDP-43 CTF in cell lysates [53]. Further and definitive evidence of exosome-mediated secretion and prion-like propagation of TDP-43 aggregates has emerged from the study of Feiler and colleagues [55]. Indeed, exosomes and MVs containing TDP-43 oligomers have shown intercellularly transmission, as well as seeding properties [55]. Finally, Iguchi and colleagues demonstrated that exosomal TDP-43 secretion is upregulated in brains from ALS patients and may induce cytoplasmic redistribution of TDP-43 in Neuro2a cells [56]. However, inhibition of exosome secretion in vivo increases TDP-43 aggregation and exacerbates the phenotype of *TDP-43* transgenic mice, suggesting that exosomes may also be implied in neuronal clearance of TDP-43 [56].

Mutations in *FUS* are responsible of a subset of cases of familial and sporadic ALS. Similarly to TDP-43, *FUS* encodes for a RBP and its mutations may prompt protein cytoplasmic mislocalization with subsequent sequestration of RNA transcripts and stress granules-like structure formation [57]. FUS immunoreactive inclusions have been found in neuronal and glial cells of patients affected by *FUS*-related fALS [57], suggesting that FUS abnormal deposition may exert neuronal toxicity and death, similarly to TDP-43 and SOD1. Recent evidence has proven that FUS colocalize with exosomes derived from wild-type and mutated *FUS*-expressing cells [58], possibly indicating that EVs might participate in cell-to-cell FUS propagation.

Hexanucleotide repeat expansion (HRE) in *C9orf72* are the most common genetic cause of both fALS and sALS. Along with inclusions of phosphorylated and ubiquitinated TDP-43, post-mortem tissues from patients with *C9orf72-*related ALS (C9-ALS) have shown the presence of DPRs derived from non-canonical translation of sense and antisense HRE-containing transcripts [59]. DPR-mediated toxicity is crucial for C9-ALS pathogenesis and is considered one of the major drivers of neuronal death [60]. DPR intercellular transmission via exosome-dependent and independent pathways has been demonstrated in spinal MNs from C9-ALS patients and DPRs transfected NSC-34 cells [61]. Although causality with neuronal death has not been reported, DPRs have been found to propagate from neurons to glial cells and viceversa, likely accounting for pathology propagation in C9-ALS [61].

**Fig. 1:**
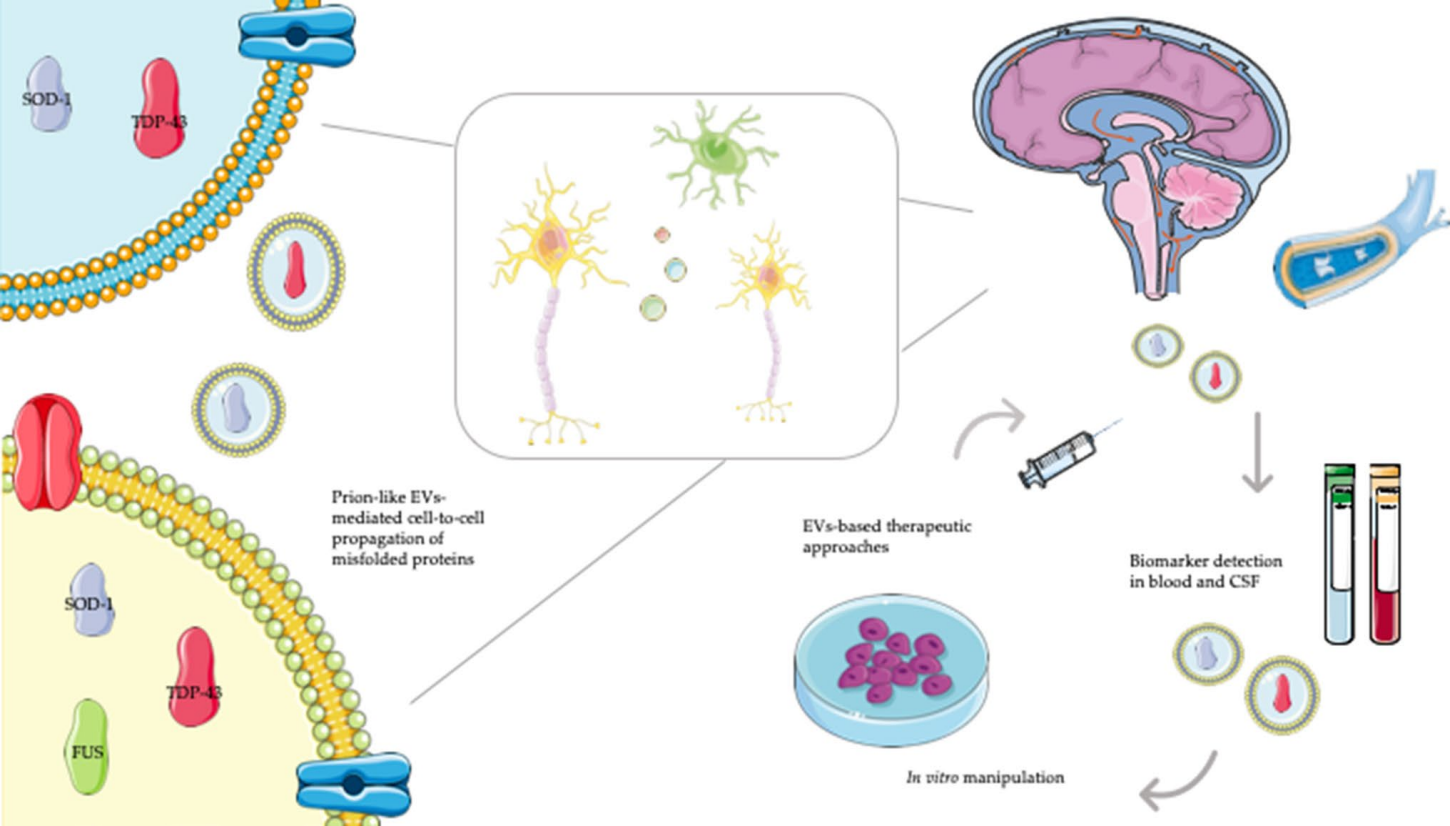
Extracellular vesicle cell transmission and their clinical applications in amyotrophic lateral sclerosis. Extracellular vesicles (EVs) mediate cell-to-cell transmission of misfolded proteins (e.g. SOD1, TDP-43 and FUS) in the central nervous system (CNS). EVs can be detectable in blood and cerebrospinal fluid (CSF) of amyotrophic lateral sclerosis (ALS) affected patients, providing diagnostic and prognostic biomarkers. After their purification from patients’ biofluids, EVs may be manipulated to accommodate proteins, non-coding RNAs or drugs, and subsequently transferred back to the same patients, serving as therapeutic vehicles

Taken together, these findings advocate the crucial contribution of EVs into the spreading of toxic proteins across CNS, and consequently in neurodegeneration (Fig. 1).

## Non-coding RNAs and EVs in ALS

As mentioned earlier, nucleic acids are one of the principal cargoes retrieved in EVs, in addition to proteins. The phospholipid bilayer surrounding EVs guarantees RNA stability in extracellular space and in biofluids, preventing degradation by ribonucleases. Other than mRNAs, transmission of non-coding RNAs provides regulation of gene expression by promoting or inhibiting translation in recipient cells, thereby providing us with valuable insight into cell functioning and disease pathogenesis.

This is particularly relevant in ALS, given that alteration in RNA homeostasis is a well-recognized pathomechanism. Indeed, some ALS-causing genes (e.g. *TARDBP* and *FUS*) are involved in RNA processing and activity and some miRNAs may alter the expression of proteins involved in ALS. Moreover, the depletion of Dicer, an enzyme crucial for miRNA biogenesis, leads to MN death [62]. Accordingly, non-coding RNAs and especially miRNAs have become promising tools useful to better understand disease pathogenesis [63, 64].

A few studies have successfully elucidated the role of miRNAs carried by EVs in modulating ALS phenotype and pathogenesis.

After previous evidence of exosome-mediated SOD1 cell-to-cell transmission as mechanism of disease spreading, Pinto and colleagues have shown that wild-type and mutated *SOD1* transfected NSC-34 MNs are able to transfer miRNA-enriched exosomes to N9 microglial cells, thus influencing their inflammatory properties [65]. Indeed, increased expression of miR-124 has been found in *SOD1*-mutated MNs and in their derived exosomes and reduction of microglial phagocytic activity, persistent Nf-KB activation, as well as upregulation of genes involved in inflammation in microglia has been observed [65]. Moreover, miR-124 has been previously correlated to astrocyte differentiation through Sox2 and Sox9 targeting [66] and to the modulation of EAAT-2 expression in astrocytes [67], which is consistent with the role of glutamate excitotoxicity in ALS pathogenesis. Given the putative role of inflammation in ALS pathogenesis, these works may provide new hints into the therapeutic modulation of microglia activation and astrocyte functioning using miRNA-containing exosomes.

Given the involvement of astrocytes in the pathogenesis of *SOD1*-related ALS, miRNA profile in exosomes released by *SOD1-*mutated astrocytes has been investigated, despite no significant difference has been found compared to wild-type *SOD1* cells [68]. Conversely, EVs released by astrocytes derived from ALS patients carrying *C9orf72* HRE present decreased levels of miR-494-3p, a negative regulator of proteins involved in axonal maintenance [69]. Restoration of miR-494-3p levels increases survival in MNs, suggesting that this miRNA may represent a promising therapeutic target [69].

Additionally, recent researches have investigated the utility of circulating miRNAs in EVs derived from ALS patients’ blood as potential biomarkers.

MiR-27a-3p, a miRNA involved in myoblast–osteoblast interaction, was detected at decreased concentrations in exosomes derived from ALS serum in comparison to healthy controls [70]. To obtain an ALS-associated miRNA signature, Saucier and colleagues have used a next-generation sequencing approach on EVs extracted from plasma of patients with ALS respect to healthy controls [71]. Differential expression of 22 miRNAs has been observed between ALS and controls and deregulation of miRNAs with relevance for ALS (i.e. miR-9-5p, miR-183-5p, miR-338-3p, miR-1246) has been identified [71]. Interestingly, miR-15a-5p and miR-193a-5p have been linked to ALS diagnosis and progression, respectively [71]. Similarly, neuronal-derived EVs extracted from plasma of ALS patients have shown deregulation of 30 miRNAs compared to healthy controls [72]. Deregulated miRNAs were involved in synaptic vesicle-related pathways and among these, 4 miRNAs have been similarly deregulated in motor cortex samples of ALS patients [72]. Using an analogous approach, a recent work identified a potential miRNA fingerprint in neural-enriched EVs: 5 miRNAs (miR-146a-5p; miR-199a-3p; miR-151a-3p; miR-151a-5p; miR-199a-5p) showed an up-regulation in ALS samples, while 3 miRNAs (miR-4454; miR-10b-5p; miR-29b-3p) were found to be downregulated in ALS compared to healthy controls [73]. Of note, dysregulation of miR-199a-3p and miR-4454 was identified also by Saucier et al. [71] and miR-199a-3p was associated to cell growth, axonal regeneration and plasticity. Moreover, miR-146a-5p, a miRNA involved in synaptic plasticity and inflammatory response, showed reduced expression in CSF of patients with ALS [74] and seems to play a relevant role in Spinal Muscular Atrophy (SMA) as well [75]. As previously mentioned, miR-146a-5p-containing EVs are able to reduce dendritic spine density and miniature synaptic currents in rats, due to inhibition of synaptotagmin1 and neuroligin1 [35]. MiR-151a-5p, which is linked to cell viability and oxidative stress response, has shown increased expression in blood and CSF samples from patients with AD [76] and PD [77].

Finally, it is worth mentioning that blood levels of miR-151a-5p, miR199a-5p, miR199a-3p, identified as upregulated in ALS by Banack and colleagues, have shown a significant correlation with clinical parameters [78].

Although still at infancy, these studies have opened the path for the utilization of EVs as elegant and innovative tools to study non-coding RNAs, possibly informative in ALS pathogenesis and diagnosis. Since the main function of miRNAs is to downregulate mRNA expression through translation block or transcript degradation, it is reasonable that recently identified miRNAs will add piece of information about disease-related genes and pathways. So far, only one study has successfully performed a RNA sequencing of mRNAs from CSF exosomes in ALS patients, identifying several candidate genes playing a role in processes related to ALS pathogenesis, such as unfolded protein response, ubiquitin–proteasome system and oxidative stress [79].

## Clinical application of EVs in ALS

### EVs as biomarkers of diagnosis and prognosis

Despite several research efforts in elucidating disease mechanisms, ALS pathogenesis is still not completely understood. Moreover, a number of experimental therapeutic approaches have been tested so far, without successful results. Non-invasive measurable and reliable biomarkers are urgently needed to achieve earlier patient diagnosis and inclusion in clinical trial, prognostic estimation and drug monitoring [80].

CNS-derived exosomes have been demonstrated to cross the BBB and enter the systemic circulation, being easily detectable in biological fluids like blood, CSF and urine. They carry specific molecular signatures, reflecting the cell status and functioning during disease condition, thus providing precious information about disease pathogenesis. Moreover, they can be easily isolated from patients’ blood and CSF through a minimally invasive maneuver [81], serving as sources of potential biomarkers in neurodegenerative disorders, including ALS.

A few studies have investigated the feasibility of this approach in biofluids from patients with ALS (Table 1). A recent work aimed to characterize number, size and content of EVs retrieved from plasma has found increased mean size in ALS patients compared to healthy controls, but no numerical difference between the two groups [82]. MVs but not exosomes of ALS patients were enriched in ALS-related proteins, including SOD1, TDP-43, pTDP-43 and FUS, confirming the findings of in vitro studies in support of EV-mediated prion-like propagation of ALS disease [82]. Similarly, TDP-43 has been retrieved in brain-derived exosomes from CSF of ALS patients, possibly reflecting in vivo the underlying neuropathological findings [54]. Moreover, leukocyte-derived MVs have been found at elevated levels in ALS patients and have shown a slight correlation with progression rate at the last visit, possibly representing biomarkers of disease progression [83].

Besides, analyses of patients’ biofluids have drawn the attention on new proteins possibly involved in ALS pathogenesis. Consistent with the role of inflammation in ALS, Chen and colleagues have found elevated levels of interleukin-6 (IL-6) in astrocyte-derived exosomes retrieved from plasma of sALS patients, in addition to a positive correlation between their levels and rate of disease progression [84]. A proteomic study performed on exosome-enriched fraction of CSF has revealed increased concentration of novel INHAT repressor (NIR), involved in nucleolar function, in ALS patients [85]; interestingly NIR has shown altered subcellular localization in MNs of affected patients.

### EVs as therapeutic tools

Other than helpful promising biomarkers, exosomes, and EVs overall, can be employed as potential therapeutics, either as targets or as vehicles of molecules relevant for cell functioning and crosstalk.

EVs are putative carriers of pathological misfolded proteins in neurodegenerative diseases; thereby, attenuating their functions might be beneficial to hamper the seed transmission and to prevent the development of neurodegeneration. In this context, several approaches targeting different stages of vesicles’ pathways could be exploited, including vesicle formation, release, trafficking and uptake [86]. These strategies have been already applied in inflammation and cancer, but their application in the field of neurodegenerative disorders should be taken into account.

The use of EVs as efficient systems to deliver molecules (e.g. proteins, genetic material or drugs) targeting cells or tissues likely represents the most innovative field of application. There are several advantages of using EVs as carriers of relevant biological molecules. First, they are highly stable in circulating system, in a way that can reach also distant targets; moreover, exosomes can cross the BBB [3] enabling the transport of cargoes to the CNS, being particularly appealing for the treatment of neurologic disorders. Then, compared to liposome- and nano-based therapies, they have cell-surface molecules and high affinity for tissues, reducing the risk of off-target effects, as well as avoiding phagocytosis or degradation by macrophages. In addition to their excellent biocompatibility and poor immunogenicity and toxicity, EVs show high availability, since they can easily be retrieved from patients’ biofluids. Ideally, after isolating exosomes from cell cultures or patients’ blood, they can be manipulated to accommodate proteins, non-coding RNAs or drugs, and subsequently transferred back to the same patients (Fig. 1).

It is worth mentioning that stem cells secrete exosomes containing cell-derived factors, which, through a paracrine effect, can modulate the surrounding biological environment. Mesenchymal stem cells (MSCs) can be easily isolated from adult and fetal tissues, including adipose tissue, and their several beneficial effects (tissue homeostasis, repair and regeneration) are at least partially mediated by exosome secretion [6]. Exosomes derived from human mesenchymal stem cells selectively promoted neurite outgrowth in cortical neuron cultures [87]. This strategy has been considered extremely attractive and valuable in the treatment of neurodegenerative disorders, including ALS.

At this purpose, exosomes isolated from murine ASCs have been shown to protect wild-type and *SOD1*-mutated MN-like NSC-34 cells from oxidative stress, increasing cell viability [88]. Similarly, they had the ability of restoring complex I activity, coupling efficiency and mitochondrial membrane potential [89]. Proteomic analysis performed on ASC-derived exosomes confirmed their putative neuroprotective role, revealing the expression of proteins involved in cell adhesion and negative regulation of apoptosis [38]. Moreover, ASC-derived exosomes were effective in reducing aggregation of SOD1 and modulating mitochondrial dysfunction in neuronal cells from *SOD1* mouse models [90]. In parallel, the use of ASCs and their exosomes as therapeutic tools in ALS has been validated in vivo as well. Systemic administration of ASCs in *SOD1*-mutated mouse delayed motor deterioration, increased MN number and neurotrophin levels into murine spinal cord [91]. A recent study demonstrated that repeated administrations of ASC-derived exosomes improved motor performance in *SOD1*-mutated mouse, protected spinal MNs from neurodegeneration, preserved neuromuscular junction, muscle morphology and functioning, and reduced glial cell activation [92]. Additionally, intranasally delivered exosomes were able to reach brains of wild-type mice and to home to injured brain regions of *SOD1*-mutated counterparts.

However, there are also some limitations hampering the translation of EVs in clinical therapies, which are mainly related to the challenge of purifying the scarce number of exosomes from the human body and of determining the best source for their retrieval. In addition, the optimization of vesicle loading and dosage is equally important to maintain the structure and the content of exosomal membranes without altering functional efficacy. Finally, the problem of how to reach selectively the target cells must be addressed.

## Concluding remarks

The development and the spreading of ALS and other neurodegenerative disorders have been linked to the cell-to-cell propagation of misfolded proteins in a prion-like fashion. Although a wide body of evidence supporting the role of EVs in this process in ALS, in vivo studies are still poor. Further, there is also contrasting evidence pointing at a beneficial role of EVs in disease pathogenesis. Nonetheless, EVs have undoubtedly added a piece of knowledge in understanding disease pathomechanisms. Moreover, the isolation of peripheral and brain-derived EVs from patients’ biofluids have provided valuable insights into their clinical application as diagnostic and prognostic biomarkers, enabling the study in vivo of molecular and pathological correlates of ALS. Despite not being so close to their use as therapeutics in the oncoming futures, the findings fulfilled so far are promising and should encourage further research efforts in this direction.

## Abbreviations

ALS: Amyotrophic Lateral sclerosis
ASCs: Adipose stem cells
BBB: Blood brain barrier
C9-ALS: C9orf72-related ALS
C9orf72: Chromosome 9 open reading frame 72
CNS: Central nervous system
CSF: Cerebrospinal fluid
CTF: C-terminal fragment
DPRs: Dipeptide repeat proteins
EVs: Extracellular vesicles
FTD: Frontotemporal dementia
FTLD: Frontotemporal lobar degeneration
FUS: Fused-in-sarcoma
HRE: Hexanucleotide repeat expansion
IL-6: Interleukin-6
iPSC: Induced pluripotent stem cell
lnRNAs: Long non-coding RNAs
miRNAs: Micro-RNAs
MN: Motor neuron
mRNAs: Messenger RNAs
MVBs: Multivesicular bodies
MVs: Microvesicles
NIR: Novel INHAT repressor
PrLD: Prion-like domain
pTDP-43: Phosphorylated-TDP-43
RBP: RNA-binding protein
ROS: Reactive oxygen species
sALS: Sporadic ALS
SOD1: Superoxide dismutase
TDP-43: TAR DNA-binding protein 43

## References

[1] Akers JC, Gonda D, Kim R (2013). Biogenesis of extracellular vesicles (EV): exosomes, microvesicles, retrovirus-like vesicles, and apoptotic bodies. J Neurooncol.

[2] Cocucci E, Meldolesi J (2015). Ectosomes and exosomes: shedding the confusion between extracellular vesicles. Trends Cell Biol.

[3] Matsumoto J, Stewart T, Banks WA, Zhang J (2017). The transport mechanism of extracellular vesicles at the blood-brain barrier. Curr Pharm Des.

[4] Kourembanas S (2015). Exosomes: vehicles of intercellular signaling, biomarkers, and vectors of cell therapy. Annu Rev Physiol.

[5] Théry C, Amigorena S, Raposo G, Clayton A (2006). Isolation and characterization of exosomes from cell culture supernatants and biological fluids. Curr Protocol Cell Biol.

[6] Lai RC, Yeo RWY, Lim SK (2015). Mesenchymal stem cell exosomes. Semin Cell Dev Biol.

[7] Greco V, Hannus M, Eaton S (2001). Argosomes: a potential vehicle for the spread of morphogens through epithelia. Cell.

[8] Turturici G, Tinnirello R, Sconzo G, Geraci F (2014). Extracellular membrane vesicles as a mechanism of cell-to-cell communication: advantages and disadvantages. Am J Physiol Cell Physiol.

[9] An K, Klyubin I, Kim Y (2013). Exosomes neutralize synaptic-plasticity-disrupting activity of Aβ assemblies in vivo. Mol Brain.

[10] Valadi H, Ekström K, Bossios A (2007). Exosome-mediated transfer of mRNAs and microRNAs is a novel mechanism of genetic exchange between cells. Nat Cell Biol.

[11] Basso M, Bonetto V (2016). Extracellular vesicles and a novel form of communication in the brain. Front Neurosci.

[12] Fauré J, Lachenal G, Court M (2006). Exosomes are released by cultured cortical neurones. Mol Cell Neurosci.

[13] Lachenal G, Pernet-Gallay K, Chivet M (2011). Release of exosomes from differentiated neurons and its regulation by synaptic glutamatergic activity. Mol Cell Neurosci.

[14] Antonucci F, Turola E, Riganti L (2012). Microvesicles released from microglia stimulate synaptic activity via enhanced sphingolipid metabolism. EMBO J.

[15] Thompson AG, Gray E, Heman-Ackah SM (2016). Extracellular vesicles in neurodegenerative disease—pathogenesis to biomarkers. Nat Rev Neurol.

[16] Fröhlich D, Kuo WP, Frühbeis C (2014). Multifaceted effects of oligodendroglial exosomes on neurons: impact on neuronal firing rate, signal transduction and gene regulation. Philos Trans R Soc Lond B Biol Sci.

[17] Upadhya R, Zingg W, Shetty S, Shetty AK (2020). Astrocyte-derived extracellular vesicles: neuroreparative properties and role in the pathogenesis of neurodegenerative disorders. J Control Release.

[18] Gosselin R-D, Meylan P, Decosterd I (2013). Extracellular microvesicles from astrocytes contain functional glutamate transporters: regulation by protein kinase C and cell activation. Front Cell Neurosci.

[19] Eitan E, Green J, Bodogai M (2017). Age-related changes in plasma extracellular vesicle characteristics and internalization by leukocytes. Sci Rep.

[20] Goedert M, Clavaguera F, Tolnay M (2010). The propagation of prion-like protein inclusions in neurodegenerative diseases. Trends Neurosci.

[21] Walsh DM, Selkoe DJ (2016). A critical appraisal of the pathogenic protein spread hypothesis of neurodegeneration. Nat Rev Neurosci.

[22] Peng C, Trojanowski JQ, Lee VM-Y (2020). Protein transmission in neurodegenerative disease. Nat Rev Neurol.

[23] Magalhães AC, Baron GS, Lee KS (2005). Uptake and neuritic transport of scrapie prion protein coincident with infection of neuronal cells. J Neurosci.

[24] Gousset K, Schiff E, Langevin C (2009). Prions hijack tunnelling nanotubes for intercellular spread. Nat Cell Biol.

[25] Asai H, Ikezu S, Tsunoda S (2015). Depletion of microglia and inhibition of exosome synthesis halt tau propagation. Nat Neurosci.

[26] Emmanouilidou E, Melachroinou K, Roumeliotis T (2010). Cell-produced alpha-synuclein is secreted in a calcium-dependent manner by exosomes and impacts neuronal survival. J Neurosci.

[27] Ngolab J, Trinh I, Rockenstein E (2017). Brain-derived exosomes from dementia with Lewy bodies propagate α-synuclein pathology. Acta Neuropathol Commun.

[28] Perez-Gonzalez R, Gauthier SA, Kumar A, Levy E (2012). The exosome secretory pathway transports amyloid precursor protein carboxyl-terminal fragments from the cell into the brain extracellular space. J Biol Chem.

[29] Saman S, Kim W, Raya M (2012). Exosome-associated tau is secreted in tauopathy models and is selectively phosphorylated in cerebrospinal fluid in early Alzheimer disease. J Biol Chem.

[30] Shi M, Liu C, Cook TJ (2014). Plasma exosomal α-synuclein is likely CNS-derived and increased in Parkinson’s disease. Acta Neuropathol.

[31] Wang Y, Balaji V, Kaniyappan S (2017). The release and trans-synaptic transmission of Tau via exosomes. Mol Neurodegener.

[32] Kfoury N, Holmes BB, Jiang H (2012). Trans-cellular propagation of Tau aggregation by fibrillar species. J Biol Chem.

[33] Garden GA, La Spada AR (2012). Intercellular (mis)communication in neurodegenerative disease. Neuron.

[34] Paolicelli RC, Bergamini G, Rajendran L (2019). Cell-to-cell communication by extracellular vesicles: focus on microglia. Neuroscience.

[35] Prada I, Gabrielli M, Turola E (2018). Glia-to-neuron transfer of miRNAs via extracellular vesicles: a new mechanism underlying inflammation-induced synaptic alterations. Acta Neuropathol.

[36] Counil H, Krantic S (2020). Synaptic activity and (neuro)inflammation in Alzheimer’s disease: could exosomes be an additional link?. J Alzheimers Dis.

[37] Marchetti B, Leggio L, L’Episcopo F (2020). Glia-derived extracellular vesicles in Parkinson’s disease. J Clin Med.

[38] Bonafede R, Brandi J, Manfredi M (2019). The anti-apoptotic effect of ASC-exosomes in an in vitro ALS model and their proteomic analysis. Cells.

[39] Neumann M, Sampathu DM, Kwong LK (2006). Ubiquitinated TDP-43 in frontotemporal lobar degeneration and amyotrophic lateral sclerosis. Science.

[40] Silverman JM, Fernando SM, Grad LI (2016). Disease mechanisms in ALS: misfolded SOD1 transferred through exosome-dependent and exosome-independent pathways. Cell Mol Neurobiol.

[41] Forsberg K, Andersen PM, Marklund SL, Brännström T (2011). Glial nuclear aggregates of superoxide dismutase-1 are regularly present in patients with amyotrophic lateral sclerosis. Acta Neuropathol.

[42] Saberi S, Stauffer JE, Schulte DJ, Ravits J (2015). Neuropathology of amyotrophic lateral sclerosis and its variants. Neurol Clin.

[43] Gomes C, Keller S, Altevogt P, Costa J (2007). Evidence for secretion of Cu, Zn superoxide dismutase via exosomes from a cell model of amyotrophic lateral sclerosis. Neurosci Lett.

[44] Basso M, Pozzi S, Tortarolo M (2013). Mutant copper-zinc superoxide dismutase (SOD1) induces protein secretion pathway alterations and exosome release in astrocytes implications for disease spreading and motor neuron pathology in amyotrophic lateral sclerosis. J Biol Chem.

[45] Grad LI, Guest WC, Yanai A (2011). Intermolecular transmission of superoxide dismutase 1 misfolding in living cells. Proc Natl Acad Sci U S A.

[46] Münch C, O’Brien J, Bertolotti A (2011). Prion-like propagation of mutant superoxide dismutase-1 misfolding in neuronal cells. Proc Natl Acad Sci U S A.

[47] Grad LI, Yerbury JJ, Turner BJ (2014). Intercellular propagated misfolding of wild-type Cu/Zn superoxide dismutase occurs via exosome-dependent and -independent mechanisms. Proc Natl Acad Sci U S A.

[48] Massenzio F, Peña-Altamira E, Petralla S (2018). Microglial overexpression of fALS-linked mutant SOD1 induces SOD1 processing impairment, activation and neurotoxicity and is counteracted by the autophagy inducer trehalose. Biochim Biophys Acta Mol Basis Dis.

[49] Silverman JM, Christy D, Shyu CC (2019). CNS-derived extracellular vesicles from superoxide dismutase 1 (SOD1)G93A ALS mice originate from astrocytes and neurons and carry misfolded SOD1. J Biol Chem.

[50] Nonaka T, Masuda-Suzukake M, Arai T (2013). Prion-like properties of pathological TDP-43 aggregates from diseased brains. Cell Rep.

[51] Rutherford NJ, Zhang Y-J, Baker M (2008). Novel mutations in TARDBP (TDP-43) in patients with familial amyotrophic lateral sclerosis. PLoS Genet.

[52] Nonaka T, Kametani F, Arai T (2009). Truncation and pathogenic mutations facilitate the formation of intracellular aggregates of TDP-43. Hum Mol Genet.

[53] Ding X, Ma M, Teng J (2015). Exposure to ALS-FTD-CSF generates TDP-43 aggregates in glioblastoma cells through exosomes and TNTs-like structure. Oncotarget.

[54] Feneberg E, Steinacker P, Lehnert S (2014). Limited role of free TDP-43 as a diagnostic tool in neurodegenerative diseases. Amyotroph Lateral Scler Frontotemporal Degener.

[55] Feiler MS, Strobel B, Freischmidt A (2015). TDP-43 is intercellularly transmitted across axon terminals. J Cell Biol.

[56] Iguchi Y, Eid L, Parent M (2016). Exosome secretion is a key pathway for clearance of pathological TDP-43. Brain.

[57] Mackenzie IR, Rademakers R, Neumann M (2010). TDP-43 and FUS in amyotrophic lateral sclerosis and frontotemporal dementia. Lancet Neurol.

[58] Kamelgarn M, Chen J, Kuang L (2016). Proteomic analysis of FUS interacting proteins provides insights into FUS function and its role in ALS. Biochim Biophys Acta Mol Basis Dis.

[59] Ash PEA, Bieniek KF, Gendron TF (2013). Unconventional translation of C9ORF72 GGGGCC expansion generates insoluble polypeptides specific to c9FTD/ALS. Neuron.

[60] Wen X, Tan W, Westergard T (2014). Antisense proline-arginine RAN dipeptides linked to C9ORF72-ALS/FTD form toxic nuclear aggregates that initiate in vitro and in vivo neuronal death. Neuron.

[61] Westergard T, Jensen BK, Wen X (2016). Cell-to-cell transmission of dipeptide repeat proteins linked to C9orf72-ALS/FTD. Cell Rep.

[62] Haramati S, Chapnik E, Sztainberg Y (2010). miRNA malfunction causes spinal motor neuron disease. Proc Natl Acad Sci U S A.

[63] Gagliardi D, Comi GP, Bresolin N, Corti S (2019). MicroRNAs as regulators of cell death mechanisms in amyotrophic lateral sclerosis. J Cell Mol Med.

[64] Hosaka T, Yamashita T, Tamaoka A, Kwak S (2019). Extracellular RNAs as biomarkers of sporadic amyotrophic lateral sclerosis and other neurodegenerative diseases. Int J Mol Sci.

[65] Pinto S, Cunha C, Barbosa M (2017). Exosomes from NSC-34 cells transfected with hSOD1-G93A are enriched in miR-124 and drive alterations in microglia phenotype. Front Neurosci.

[66] Zhou F, Guan Y, Chen Y (2013). miRNA-9 expression is upregulated in the spinal cord of G93A-SOD1 transgenic mice. Int J Clin Exp Pathol.

[67] Morel L, Regan M, Higashimori H (2013). Neuronal exosomal miRNA-dependent translational regulation of astroglial glutamate transporter GLT1. J Biol Chem.

[68] Jovičić A, Gitler AD (2017). Distinct repertoires of microRNAs present in mouse astrocytes compared to astrocyte-secreted exosomes. PLoS ONE.

[69] Varcianna A, Myszczynska MA, Castelli LM (2019). Micro-RNAs secreted through astrocyte-derived extracellular vesicles cause neuronal network degeneration in C9orf72 ALS. EBioMedicine.

[70] Xu Q, Zhao Y, Zhou X (2018). Comparison of the extraction and determination of serum exosome and miRNA in serum and the detection of miR-27a-3p in serum exosome of ALS patients. Intractable Rare Dis Res.

[71] Saucier D, Wajnberg G, Roy J (2019). Identification of a circulating miRNA signature in extracellular vesicles collected from amyotrophic lateral sclerosis patients. Brain Res.

[72] Katsu M, Hama Y, Utsumi J (2019). MicroRNA expression profiles of neuron-derived extracellular vesicles in plasma from patients with amyotrophic lateral sclerosis. Neurosci Lett.

[73] Banack SA, Dunlop RA, Cox PA (2020). An miRNA fingerprint using neural-enriched extracellular vesicles from blood plasma: towards a biomarker for amyotrophic lateral sclerosis/motor neuron disease. Open Biol.

[74] Waller R, Wyles M, Heath PR (2017). Small RNA sequencing of sporadic amyotrophic lateral sclerosis cerebrospinal fluid reveals differentially expressed miRNAs related to neural and Glial activity. Front Neurosci.

[75] Sison SL, Patitucci TN, Seminary ER (2017). Astrocyte-produced miR-146a as a mediator of motor neuron loss in spinal muscular atrophy. Hum Mol Genet.

[76] Leidinger P, Backes C, Deutscher S (2013). A blood based 12-miRNA signature of Alzheimer disease patients. Genome Biol.

[77] dos Santos MCT, Barreto-Sanz MA, Correia BRS (2018). miRNA-based signatures in cerebrospinal fluid as potential diagnostic tools for early stage Parkinson’s disease. Oncotarget.

[78] Raheja R, Regev K, Healy BC (2018). Correlating serum micrornas and clinical parameters in amyotrophic lateral sclerosis. Muscle Nerve.

[79] Otake K, Kamiguchi H, Hirozane Y (2019). Identification of biomarkers for amyotrophic lateral sclerosis by comprehensive analysis of exosomal mRNAs in human cerebrospinal fluid. BMC Med Genomics.

[80] Gagliardi D, Meneri M, Saccomanno D (2019). Diagnostic and prognostic role of blood and cerebrospinal fluid and blood neurofilaments in amyotrophic lateral sclerosis: a review of the literature. Int J Mol Sci.

[81] Hornung S, Dutta S, Bitan G (2020). CNS-derived blood exosomes as a promising source of biomarkers: opportunities and challenges. Front Mol Neurosci.

[82] Sproviero D, La Salvia S, Giannini M (2018). Pathological proteins are transported by extracellular vesicles of sporadic amyotrophic lateral sclerosis patients. Front Neurosci.

[83] Sproviero D, La Salvia S, Colombo F (2019). Leukocyte derived microvesicles as disease progression biomarkers in slow progressing amyotrophic lateral sclerosis patients. Front Neurosci.

[84] Chen Y, Xia K, Chen L, Fan D (2019). Increased interleukin-6 levels in the astrocyte-derived exosomes of sporadic amyotrophic lateral sclerosis patients. Front Neurosci.

[85] Hayashi N, Doi H, Kurata Y (2019). Proteomic analysis of exosome-enriched fractions derived from cerebrospinal fluid of amyotrophic lateral sclerosis patients. Neurosci Res.

[86] Samanta S, Rajasingh S, Drosos N (2018). Exosomes: new molecular targets of diseases. Acta Pharmacol Sin.

[87] Lopez-Verrilli MA, Caviedes A, Cabrera A (2016). Mesenchymal stem cell-derived exosomes from different sources selectively promote neuritic outgrowth. Neuroscience.

[88] Bonafede R, Scambi I, Peroni D (2016). Exosome derived from murine adipose-derived stromal cells: neuroprotective effect on in vitro model of amyotrophic lateral sclerosis. Exp Cell Res.

[89] Calabria E, Scambi I, Bonafede R (2019). ASCs-exosomes recover coupling efficiency and mitochondrial membrane potential in an in vitro model of ALS. Front Neurosci.

[90] Lee M, Ban J-J, Kim KY (2016). Adipose-derived stem cell exosomes alleviate pathology of amyotrophic lateral sclerosis in vitro. Biochem Biophys Res Commun.

[91] Marconi S, Bonaconsa M, Scambi I (2013). Systemic treatment with adipose-derived mesenchymal stem cells ameliorates clinical and pathological features in the amyotrophic lateral sclerosis murine model. Neuroscience.

[92] Bonafede R, Turano E, Scambi I (2020). ASC-exosomes ameliorate the disease progression in SOD1(G93A) murine model underlining their potential therapeutic use in human ALS. Int J Mol Sci.

